# Physician knowledge, attitudes, and perceptions of antibiograms: a pre-implementation study in southern Sri Lanka

**DOI:** 10.1017/ash.2025.10124

**Published:** 2025-11-20

**Authors:** Lorenna C. Garcia-Bochas, Sherine Nanayakkara, Perla Medrano, Ajith Nagahawatte, C. Lakmal Fonseka, Armstrong Obale, Bhagya Piyasiri, Ruvini Kurukulasooriya, Madureka Premamali, Ganga Moorthy, Lana Abusalem, Melissa H. Watt, Christopher W. Woods, Truls Østbye, Champica Bodinayake, L. Gayani Tillekeratne

**Affiliations:** 1 Duke University, Durham, NC, USA; 2 Duke Global Health Institute, Durham, NC, USA; 3 Duke-Ruhuna Collaborative Research Centre, Faculty of Medicine, University of Ruhuna, Galle, Sri Lanka; 4 Faculty of Medicine, University of Ruhuna, Galle, Sri Lanka; 5 National Hospital Galle, Galle, Sri Lanka; 6 University of Utah, School of Medicine, Department of Population Health Sciences, Salt Lake City, UT, USA

## Abstract

**Objective:**

The purpose of this study is to explore physicians’ knowledge, attitudes, and perceptions toward antibiograms and identify perceived barriers and facilitators to their implementation in a low-resource setting in Sri Lanka.

**Design:**

A qualitative study utilizing semi-structured interviews and thematic analysis.

**Setting:**

A public tertiary care hospital in southern Sri Lanka.

**Participants:**

Thirty physicians working in pediatric and adult medical wards were purposively sampled and interviewed between June and August 2023.

**Results:**

Most physicians had limited prior knowledge or experience with antibiograms. However, after receiving a brief explanation, 29 out of 30 participants expressed strong support for implementing antibiograms, citing potential benefits such as improved antibiotic prescribing, reduced antimicrobial resistance (AMR), and enhanced patient outcomes. Approximately one-third of participants expressed concerns about feasibility due to time constraints, limited laboratory infrastructure, and personnel shortages. Participants recommended delivering antibiogram training through small-group sessions led by a multidisciplinary team. Thematic analysis identified three core themes: (1) limited baseline knowledge of antibiograms, (2) perceived clinical value and enthusiasm for implementation, and (3) barriers related to healthcare system constraints.

**Conclusions:**

Physicians in this LMIC setting demonstrated high interest in using antibiograms to guide empiric antibiotic therapy and address AMR. Despite logistical and infrastructural challenges, tailored training and stakeholder engagement may facilitate the successful development and use of antibiograms in similar resource-limited settings.

## Introduction

Antimicrobial resistance (AMR) is a significant global health threat, particularly in low- and middle-income countries (LMICs).^1^ AMR occurs when microbes evolve to resist medications, leading to drug inefficiency, prolonged illness, increased healthcare costs, and higher transmission and mortality rates.^2^ In LMICs, AMR is often worse due to the higher burden of infections, easy access to antimicrobials without prescriptions, weak regulations, and limited resources like microbiology laboratories and diagnostic tools.^3^

Misuse of antimicrobials is a key driver of AMR.^2^ Antimicrobial stewardship programs (ASPs) improve the rational use of antimicrobials and help decrease resistance.^4^ The World Health Organization (WHO) recommends ASPs, involving a team of infectious diseases physicians, pharmacists, nurses, and microbiologists, to enhance antibiotic use in acute care facilities.^5^

An essential component of an ASP’s toolkit is the hospital antibiogram, which summarizes local susceptibility trends of common bacterial or fungal organisms.^6^ Data for building an antibiogram is obtained from the microbiology laboratory and analyzed to show the number of isolates within a specific time frame, usually six months to one year.^6^ It displays percentages of microbial isolates susceptible to available antimicrobial agents, guiding prescribing when the microbiologic cause or susceptibility is unknown.^6^

Antibiograms have improved empirical antibiotic prescribing in high-income countries.^7,8^ In LMICs, antibiograms are one strategy where treatment decisions can be enhanced due to the frequent lack of specific culture data.^9^ However, antibiograms and the necessary technical expertise, microbiologic data, and personnel are scarce in low-resource settings, with limited studies on their development and implementation in LMICs.^10,11^

Sri Lanka is a South Asian country with a lower-middle-income economy where AMR is a prominent public health issue. High rates of antibiotic use at first contact area well-recognized issue in Sri Lanka.^12^

There is a lack of literature regarding antibiogram development and implementation in Sri Lanka. Additionally, a significant barrier to producing meaningful antibiograms in LMICs is the lack of consistent and reliable microbiological culture data. In this qualitative study, we assessed Sri Lankan physicians’ knowledge, attitudes, and practices on antibiograms to inform future hospital antibiogram implementation strategies that are feasible within resource-constrained settings.

## Methods

### Setting

This qualitative study was conducted from June to August 2023 at the largest (1800-bed) public tertiary care hospital in Southern Province, Sri Lanka. All medications and care in the inpatient and outpatient setting is provided free of charge in public hospitals. Currently, no antibiograms have been developed or implemented at this hospital.

### Context

In Sri Lanka’s public healthcare system, all medical care, diagnostic testing, and medications are provided free of charge to patients within government hospitals. However, medication stockouts may occur in public hospitals, and patients may need to purchase antibiotics out of pocket from private pharmacies when hospital supplies are unavailable. Additionally, laws prohibit the purchase of antibiotics over the counter; however, antibiotics are widely available in private pharmacies without a prescription, increasing variability in patient access and adherence. Physicians often consider cost when prescribing, as they are very cognizant of costs to the government system and consider it their responsibility to optimize the use of limited resources. In addition, physicians want to ensure that patients can afford medications if they need to obtain them outside the hospital system.

### Laboratory procedures

At the study hospital, cultures and antibiotic susceptibility testing are available through the hospital laboratory for all admitted patients at no charge; however, resource limitations may cause delays or result in selective testing. The laboratory follows standard protocols for culturing clinical specimens. Antibiotic susceptibility testing is primarily performed manually using the Kirby-Bauer disk diffusion method, which is interpreted according to the guidelines of the Clinical and Laboratory Standards Institute (CLSI). Automated systems are not routinely used due to cost constraints, except for the identification of organisms from blood cultures. These practices affect both the availability and timeliness of susceptibility results, which can impact empiric prescribing decisions.

### Participants

Participants were physicians (adult and pediatric medicine) practicing in the hospital’s pediatric or adult medical wards. Our research team recruited participants using convenience sampling through in-person outreach during working hours in the medical and pediatric wards. A total of 31 physicians were approached, of whom 31 consented and participated in the study, with one later being withdrawn due to the physician being added to the study team.

### Ethical approval

All participants provided written informed consent in English, the language commonly used in the professional medical setting. This research study was approved by the Ethical Review Committee of the Faculty of Medicine, University of Ruhuna (Sri Lanka), and the Duke University Institutional Review Board (USA). While the approvals did not explicitly mention the use of individual quotes, the inclusion of anonymized participant quotations is a standard and ethically accepted practice in qualitative research to support thematic findings. No identifying information was linked to any quote, and all quotes are labeled with participant codes to ensure confidentiality.

### Interview guide

The research team, comprised of Sri Lankan and US investigators, developed the interview guide to explore knowledge of antibiograms, attitudes toward future use of antibiograms, and training that would be beneficial before antibiogram implementation. Participants were asked 30 questions in five sections (Supplementary Figure 1).

The interview guide included examples of follow-up probing questions. In addition to open-ended questions, five questions were asked using a 5-point Likert scale (Strongly Agree, Agree, Neutral, Disagree, Strongly Disagree). Research team members pretested the guide with two pilot interviews to enhance comprehension and flow and made adjustments accordingly.

### Interview procedures

Trained research team members conducted face-to-face, semi-structured interviews in English in private areas of the hospital. Each interview lasted approximately 20 – 30 minutes. There search team collected sociodemographic information from all participants, including name, age, gender, medical specialty, working position, and medical school graduation year. Interviews were audio recorded and recordings were transcribed verbatim.

### Data analysis

Thematic content analysis was used to summarize the interview data.^13^ First, thematic codes and a codebook based on the structure and content of the interview guide were created. Then, two research team members (LGB and SN) independently reviewed interviews using NVivo software (Release 1.0, 2020) to identify themes. Any emerging themes, such as patterns of meaning that recurred across multiple interviews and were not initially identified in the codebook, were noted. Any discrepancies in coding were discussed between the two team members and resolved. Next, analytic summaries of the data were created: one for adult physicians and one for pediatricians. Topics relevant to each inquiry domain were summarized (e.g., barriers to antibiogram development and opportunities for intervention), and representative quotes from transcripts were identified.

## Results

### Study cohort and identified domains and themes

A total of 31 interviews were conducted, but only 30 were included in the data analysis because one physician later joined the study team as staff. The ages of participants ranged from 28 to 60 years, with most physicians in their 30s (18/30, 60.0%; Table 1). A total of 20/30 (66.7%; Table 1) participants were male. Among participants, 7 were Consultant Physicians (attending-level doctors), 6 were Senior Registrars, 10 were Registrars, and 7 were House Officers. In the Sri Lankan medical system, Registrars are physicians undergoing postgraduate training in internal medicine or pediatrics (roughly equivalent to U.S. residents), while Senior Registrars are in more advanced training, comparable to senior residents or fellows.

**Table 1.** Sociodemographic characteristics of physicians interviewed in the tertiary care facility in Southern Province, Sri Lanka

Variable	Characteristics	Frequency (N = 30)	Percent
Age	20s	3	10.0%
	30s	18	60.0%
	40s	3	10.0%
	50s	5	16.7%
	60s	1	3.3%
Sex	Male	20	66.7%
	Female	10	33.3%
Specialty	Pediatrics	11	36.7%
	General/ Internal Medicine	12	40.0%
	Non-Specialty	1	3.3%
	No answer	6	20.0%
Classification	Consultant physician	7	23.3%
	House Officer/Senior House Officer	7	23.3%
	Registrar	10	33.3%
	Senior Registrar	6	20.0%
Years practicing medicine	0 – 5	7	23.3%
	6 – 10	11	36.7%
	11 – 15	3	10.0%
	16 – 20	2	6.7%
	21+	7	23.3%

The analysis identified themes across five significant domains: 1) Knowledge of antibiograms, 2) Use of an antibiogram when treating a patient with a urinary tract infection (UTI), hypothetical clinical scenario with a sample antibiogram), 3) Perspectives regarding antibiograms, 4) Attitudes towards antibiogram training and implementation, and 5) Attitudes towards the development of an antibiogram for this facility.

Supplementary Table 1 presents a table of quotes that summarize the themes that emerged. Each theme is detailed below. The responses from both pediatricians and adult physicians are integrated within each thematic description.

#### Domain 1: Knowledge of antibiograms

##### Overall conclusion

Physicians demonstrated limited knowledge of antibiograms, with few having practical exposure or a clear understanding of their purpose or interpretation.

##### Summary of findings

*Limited awareness and exposure*

Twelve of the 30 physicians reported some familiarity with the concept of an antibiogram. Still, most had never seen one in practice, and only one physician demonstrated a comprehensive understanding of its clinical application.

*Superficial theoretical knowledge*

For those aware of antibiograms, knowledge was often limited to brief mentions of antibiograms during medical school or examinations, lacking practical experience in interpreting or integrating them into prescribing decisions.

*Antibiogram unavailability*

Many physicians assumed antibiograms were not available in their hospitals or the broader healthcare system, which discouraged them from seeking or using them in clinical decision-making.

#### Domain 2: Use of an antibiogram in a hypothetical clinical scenario

Overall conclusion physicians’ empiric antibiotic choices for UTIs varied widely, often leaning toward broad-spectrum agents inconsistent with guideline-based thresholds; however, access to an antibiogram improved alignment with recommended susceptibility rates. Decision-making was shaped by themes of patient-centered considerations, resource constraints, and flexibility in applying guidelines.

##### Summary of findings

*Patient-specific considerations*

Physicians weighed factors such as patient age, formulation palatability, side effect profiles (e.g., diarrhea), and potential complications, particularly when treating pediatric versus adult patients. Pediatricians emphasized child-friendly formulations, while adult physicians focused more on patient stability and uncomplicated infection presentations.

*Cost and availability constraints*

Even when antibiogram data suggested alternative options, many physicians considered the affordability of oral antibiotics and the practical limitations of IV-only agents, which often require hospitalization and impose additional costs on patients when medications were unavailable in hospital pharmacies.

*Guideline flexibility and adherence*

While most physicians expressed willingness to adjust empiric therapy to meet an 80% susceptibility threshold, several highlighted the importance of clinical judgment, citing circumstances where rigid adherence might be impractical due to limited oral options or patient-specific factors.

#### Domain 3: Perspectives regarding antibiograms

#### Overall conclusion

Physicians viewed antibiograms positively as practical and generally easy-to-understand tools for guiding therapy, and they offered specific, actionable recommendations to enhance their relevance and useability in practice.

#### Summary of findings

Comprehensibility and initial learning curve. Most physicians found the antibiogram format clear and straightforward once it was explained, although a few noted initial difficulties in interpreting it, which improved with review and discussion.

*Content enhancement recommendations*

Pediatricians suggested adding commonly prescribed pediatric antibiotics (e.g., cephalexin, cefuroxime) and including information on antibiotic spectra (Gram-positive versus Gram-negative), regional resistance patterns, and drug costs. Adult physicians emphasized the need to expand oral antibiotic options relevant to outpatient care (e.g., ampicillin, ciprofloxacin) and to ensure that key intravenous (IV) antibiotics, such as imipenem, were represented in inpatient decision-making.

*Improved presentation and specificity*

Both pediatric and adult physicians recommended incorporating visual aids, such as color-coding and separation of oral versus IV antibiotics, to improve clarity. They also suggested tailoring antibiograms with sample-specific data (e.g., urine versus blood isolates) to enhance clinical relevance. Pediatricians additionally highlighted uncertainty over whether current antibiograms adequately address pediatric needs, underscoring the importance of age-specific susceptibility information.

*Additional perspectives on benefits and concerns*

Physicians noted several broader benefits of implementing antibiograms, including potential reductions in hospital-acquired infections through improved empiric therapy and enhanced confidence in prescribing decisions, especially for junior staff. Some participants also expressed concerns that improper interpretation of antibiogram data could lead to overtreatment or under-treatment, particularly if clinicians lacked sufficient training. Others highlighted the importance of regular updates to antibiograms to avoid outdated guidance leading to ineffective empiric choices.

#### Domain 4: Attitudes towards antibiogram training and implementation

##### Overall conclusion

Physicians strongly supported antibiogram training and identified clear preferences for interactive, practical, and multidisciplinary instruction to promote effective implementation in clinical practice.

##### Summary of findings

*High receptiveness to training*

Almost all physicians expressed strong agreement on the need for antibiogram training to improve their knowledge and patient care practices, with only one participant remaining neutral.

*Preferred training formats*

Most physicians favored interactive approaches, with smaller group sessions cited for promoting engagement and discussion, while some preferred large-group sessions to address hospital-wide issues such as antibiotic availability and cost. Virtual options, such as Zoom, were also valued for their convenience and accessibility, with several physicians highlighting the benefit of including all medical staff, from interns to consultants, to ensure a shared understanding.

*Preferred instructors and multidisciplinary involvement*

Many physicians recommended involving microbiologists as lead trainers, recognizing their expertise in antibiotic susceptibility patterns. Others advocated for a collaborative approach that incorporated clinicians, consultants, and infection control nurses to ensure training remained practical, relevant, and widely accepted. A smaller number of participants emphasized clinician-led components to connect training directly to patient-centered care.

#### Domain 5: Attitudes toward development of an antibiogram for this facility

##### Overall conclusion

Physicians strongly supported the development of a facility-specific antibiogram, recognizing its potential to improve empiric prescribing and reduce AMR, while acknowledging practical barriers and emphasizing the need to integrate clinical judgment.

##### Summary of findings

*Precevied benefits and importance*

All physicians agreed or strongly agreed on the importance of developing an antibiogram, citing benefits such as reducing inappropriate antibiotic use, curbing resistance, improving patient outcomes, and containing costs by minimizing unnecessary prescriptions. High patient volumes were also seen as reinforcing the need for antibiogram-guided prescribing to manage resistance risks effectively.

*Considerations for balanced implementation*

Physicians emphasized the importance of combining antibiogram guidance with clinical judgment to prevent overreliance on susceptibility data, which could result in either overtreatment or under-treatment. Many stressed that antibiograms should complement, not replace, physicians’ clinical assessments and emphasized the need for regular updates to maintain accuracy and relevance.

*Anticipated barriers*

Resource limitations, including a lack of laboratory capacity, staff, and information technology infrastructure, were identified as significant obstacles to developing and maintaining an antibiogram. Heavy clinician workloads were also viewed as potential challenges to uptake and use. Some physicians noted possible resistance from senior staff accustomed to relying on personal experience. At the same time, difficulties in obtaining high-quality samples and complete documentation were frequently mentioned as practical impediments to generating reliable antibiogram data.

## Discussion

This study identified gaps in Sri Lankan physicians’ understanding of antibiogram interpretation and application but also noted their universal recognition of these tools as essential for optimizing antimicrobial use and enhancing patient outcomes. Physicians acknowledged the role of antibiograms in combating antibiotic resistance by improving prescribing practices, but they emphasized the need for better training to use them effectively.

The responses to the UTI case scenarios revealed important insights when compared to standard prescribing practices. International and national guidelines (e.g., World Health Organization, Infectious Diseases Society of America, and Sri Lankan Ministry of Health) recommend selecting empiric antibiotics based on local susceptibility patterns, often using a threshold of ≥ 80% susceptibility to guide effective treatment. In our study, many physicians initially selected broad-spectrum antibiotics such as ciprofloxacin, cefuroxime, and amoxicillin-clavulanate when they did not have access to susceptibility data—choices that may not align with guideline recommendations due to lower resistance thresholds or concerns about overuse. When provided with an antibiogram, physicians’ choices shifted toward antibiotics with higher susceptibility (e.g., meropenem, imipenem, nitrofurantoin), reflecting improved alignment with guideline-based practices. However, physicians still considered factors such as drug availability, cost, patient age, and route of administration. These findings illustrate both the potential impact of antibiograms on improving guideline-concordant prescribing and the practical constraints that influence decision-making in resource-limited settings.

Antibiogram knowledge and access were low among physicians, similar to what has been shown in US, Latin American, and South Asian studies.^14–16^ Among US medical residents, Cooper et al. found that only 24 out of 42 (57%) knew what an antibiogram was and provided a correct description of at least one attribute.^14^ In Pakistan, Atif et al. found that doctors’ inadequate knowledge of antibiotic stewardship programs, a lack of hospital antibiograms, and a lack of regulations for antibiotic use contributed to irrational antibiotic prescribing and antibiotic resistance.^16^ In Latin America, 51% of healthcare workers lacked access to antibiograms and 34% lacked access to local guidelines.^15^

Although physicians had limited knowledge of antibiograms, they acknowledged their value in understanding local susceptibility patterns, guiding antibiotic selection, and improving patient outcomes. These findings are consistent with those from other studies.^22,23^ Khatri et al. found that Australian healthcare professionals recognized antibiograms as valuable tools for strengthening empirical antibiotic prescribing and reducing ineffective prescriptions, highlighting the importance of physician awareness and perceptions similar to those identified in our study.^17^ In contrast, Wali et al. reported that while healthcare providers in Saudi Arabia had good access to and frequently used antibiograms, several barriers hindered their consistent application, including lack of expertise, technological infrastructure, and funding.^18^

Our findings suggest variability in physicians’ willingness to adhere strictly to standard susceptibility thresholds. Although we did not systematically examine associations with clinicians’ levels of experience or understanding of antibiograms, exploring these factors in future studies could inform targeted implementation strategies, such as customizing training or support based on experience level or baseline familiarity with antibiograms.

Physicians expressed willingness for antibiogram training, recognizing its potential to enhance antibiotic prescribing and patient care. A study in the US found that 47% wanted more education on interpreting antibiograms and guiding empiric antibiotic selection, while 98% sought additional local antibiotic resistance resources.^19^

Antibiograms inform antibiotic therapy but are less prevalent in LMICs due to limited healthcare capacity, inadequate surveillance, and resource constraints. These issues lead to outdated antibiograms, as high patient volumes prevent timely updates. Communication gaps between labs and clinicians further hinder dissemination. LMICs need targeted investments in laboratory infrastructure, capacity building, and updated antibiogram processes to overcome these barriers. This study highlights the need for antibiograms and educational programs in LMICs dealing with AMR, filling a gap often overlooked in literature focused on high-income countries.

This study’s strengths include the involvement of both pediatric and adult physicians at a tertiary care hospital. The clinical scenario and sample antibiogram offered practical insights into physicians’ understanding and implementation. Limitations of this study include the use of convenience sampling and a small sample size, both of which can introduce selection bias and limit the generalizability of the findings beyond the study setting. Although data saturation was achieved among interviewed physicians, the exclusive focus on physicians excluded perspectives from other key stakeholders—such as pharmacists and microbiologists—whose insights could have provided a more comprehensive understanding of barriers to antibiogram implementation in diverse healthcare environments, including smaller institutions and community settings. These factors should be taken into account when interpreting and applying the results of this study to other contexts.

Our findings are consistent with previous studies in Sri Lanka, such as the work by Gunasekara, Rathnayaka, and Vidanage (2022) at the National Cancer Institute, which reported similar limitations in the generation and use of antibiograms due to inconsistent culture testing and laboratory constraints.^20^ These shared challenges highlight systemic issues that must be addressed to improve the utility of antibiograms for guiding antibiotic therapy in Sri Lankan hospitals.

In conclusion, our study of physicians at a tertiary care hospital in the Southern Province of Sri Lanka revealed that knowledge of antibiograms was generally low, yet enthusiasm for their implementation was high. Despite concerns about time and resource constraints, there was a strong overall willingness to engage in training on the use of antibiograms. These findings provide valuable insights into the development and deployment of antibiograms in Sri Lanka and similar LMICs.

## Supporting information

10.1017/ash.2025.10124.sm001 Garcia-Bochas et al. supplementary material 1 Garcia-Bochas et al. supplementary material

10.1017/ash.2025.10124.sm002 Garcia-Bochas et al. supplementary material 2 Garcia-Bochas et al. supplementary material

## Supplementary material

The supplementary material for this article can be found at https://doi.org/10.1017/ash.2025.10124.
