# Supplementary material for: Physician knowledge, attitudes, and perceptions of antibiograms: a pre-implementation study in southern Sri Lanka
Source: Antimicrob Steward Healthc Epidemiol. 2025 Nov 20;5(1):e309. doi: 10.1017/ash.2025.10124 (PMC12645232; doi:10.1017/ash.2025.10124)
Supplement: Garcia-Bochas et al. supplementary material 1 — Garcia-Bochas et al. supplementary material [file S2732494X25101241sup001.pdf]

Interview Questionnaire (20-30 min)

The aim of this project is to study how physicians at this hospital make decisions about prescribing antibiotics and how they feel about implementing tools that could help improve the prescribing of antibiotics in the future. We want to identify resources you currently use to prescribe antibiotics and any barriers you face to appropriately prescribing antibiotics. We also would like to present a sample tool called an antibiogram and learn how having this diagnostic tool at your hospital may inform your prescribing decisions. Lastly, we would like to know your thoughts about receiving future training to learn how to use this tool effectively.

This interview will be audio-recorded, transcribed verbatim, and analyzed by our team of researchers. The tape recording will be deleted at the conclusion of the study. All your information will remain confidential, and your name will not be linked to the recording or transcript of your interview. You have the right to withdraw from the study at any point until the study has been published.

- Do you have any questions about the information I presented to you?
- Could you please sign the consent form?

Thank you for participating in our study. We truly appreciate it.

<u>Demographics</u>

1. Please tell me your age, gender, medical specialty, working position, and medical school graduation year.

<u>Antibiotic Prescribing Practices</u>

2. If you decide to prescribe an antibiotic for a patient, what factors do you consider when selecting a specific antibiotic?
3. On average, how many antibiotics do you have to choose from?
4. Are there specific antibiotics that most physicians prescribe in this hospital? What about in your ward?
5. How do you decide which antibiotic to prescribe when culture data are not available?
   - Does this vary based on the type of infection? For example, when treating pneumonia vs a urinary tract infection?

<u>Knowledge and Attitudes Towards Antimicrobial Resistance</u>

6. What concerns, if any, do you have regarding adverse effects or negative consequences when prescribing antibiotics?
7. What kind of organisms have shown the most resistance to antibiotics in the area you serve? Have you observed any resistance patterns in this hospital?
8. How does knowledge of resistance in this area or hospital influence your prescribing patterns?

Knowledge of Antibiograms

Now I'm going to give you a hypothetical scenario, and I'd like your thoughts. There is no right or wrong answer.

9. In the last year, when all *E. coli* isolates collected at THK were tested against the antibiotic ciprofloxacin, 50% of the isolates were susceptible to ciprofloxacin. How comfortable would you feel using ciprofloxacin for a patient presenting with an infection due to *E. coli*?
    - Very uncomfortable, uncomfortable, neutral, comfortable, or very comfortable.
    - Please explain why you chose that answer.

10. What minimum percentage of antibiotic susceptibility to ciprofloxacin would you feel comfortable with when treating an *E. coli* infection?
    - 30%, 50%, 80%, or 95%.
    - Please explain why you chose that answer.

Stage 1: Scenario without antibiogram

We would like to present a scenario and ask you how you would prescribe antibiotics.

* Present one of the following scenarios:

- *Adult example:*
  A 25-year-old healthy female presents to the emergency department with increased urinary frequency and burning during urination. She has not had blood in her urine, fevers, flank pain, or vaginal discharge. She has no prior history of infections and is on no other medications. She is afebrile with normal vital sign parameters. On the physical exam, she has mild abdominal discomfort with palpation. Urine analysis shows > 50 white blood cells. Urine culture shows Gram-negative rods.

- *Pediatric example:*
  A 2-year-old healthy female presents to the emergency department with fever and increased urinary frequency. Her mother has not noticed cough, congestion, vomiting, diarrhea, or blood in her urine. She has no prior history of infections, was born full-term without complications, and is on no other medications. She is febrile to 38.5°C without hypotension or tachycardia. On the physical exam, she has mild abdominal discomfort with palpation with normal tympanic membranes, no upper respiratory symptoms, and normal lung exam. Urine analysis shows > 50 white blood cells. Urine culture shows Gram-negative rods.

11. In this situation, without further information about susceptibilities, what would you typically choose as your empiric antibiotic?

12. Why would you choose that antibiotic?
    - Are there any other antibiotics you may consider using for this scenario?
    - What factors did you consider to rule out the other antibiotics?

Stage 2: Scenario with antibiogram but no threshold values

13. Have you ever heard of an antibiogram? If so, can you describe what it is? Have you ever used one before?

Antibiograms are tools that help providers select the most appropriate empiric antibiotic treatment when microbiological data are not yet available. They are used to monitor local susceptibility trends and track changes in antimicrobial resistance. Antibiograms are created using microbiology data from the hospital's microbiology laboratory. When antibiograms are first made, they show at least six months of data. It is recommended that they eventually reflect one year of data and are updated yearly.

Presenting sample antibiogram - This is a sample antibiogram. The first column lists the organisms organized by Gram-negative or Gram-positive results. The second column shows the total number of isolates from patients in your facility within a specific timeframe (e.g., six months or a year). The next columns show the antibiotics tested and the susceptibility percentages for the organisms listed. A score of 0 suggests that all isolates were resistant and 100 indicates that all isolates were susceptible. To use an antibiogram, you first locate the organism of interest and select the appropriate empiric antibiotic based on the susceptibility scores.

14. Do you have any questions about the antibiogram or what information is presented in it?

15. How do you think the information presented in antibiograms might inform your prescribing decisions?

16. Let's go back to the UTI scenario. What antibiotics might you select with the additional information from the antibiogram?

17. Is the information presented in the antibiogram easy to understand?

18. Do you have recommendations for additional information that could be included in the antibiogram?

Stage 3: Scenario with threshold values

19. In standard practice in the U.S., the threshold that is generally selected is 80% susceptibility in terms of when an antibiotic is recommended to be used empirically against an organism. Now, going back to the scenario, would knowledge of this threshold alter your decision change?

    - Why or why not?

20. Given this additional information, what antibiotic would you prescribe empirically?

Receptiveness Towards Antibiogram Training and Implementation Feasibility

21. *Please select the appropriate response about receiving training on antibiograms:*
    I would be open to receiving training and advice regarding antibiogram use in prescribing practices.

    - Strongly disagree, disagree, neither agree nor disagree, agree, or strongly agree

22. What form of training would you prefer? Are there any specific components you would like the training to include? Who would you like the training to be delivered by?

23. If an antibiogram was developed at your facility, would you like the information to be divided by syndrome? (If they do not comprehend, prompt with the following — for example, by urinary tract infection or pneumonia?)

    - Yes or no? If yes, what syndromes?

24. How often would you want an antibiogram to be updated in order for you to feel comfortable using it in your prescribing decisions?

    - Every six months, every year, or other?
    - Please explain your answer.

25. Is there any other information that would be helpful when making empiric prescribing decisions?

26. *Please select the appropriate response about utilizing antibiograms:*
    I would be open to using an antibiogram every time I prescribe antibiotics.
    - Strongly disagree, disagree, neither agree nor disagree, agree, or strongly agree
    - Please explain your answer.

27. *Please select the appropriate response about implementing antibiograms:*
    a. It is important to develop an antibiogram for this facility to reduce inappropriate antibiotic use.
    - Strongly disagree, disagree, neither agree nor disagree, agree, or strongly agree
    - Please explain your answer.

    b. It would be feasible for my facility to have a system in which antibiograms are developed and updated annually.
    - Strongly disagree, disagree, neither agree nor disagree, agree, or strongly agree
    - Please explain your answer.

28. Do you think there could be negative consequences in implementing an antibiogram in this facility? Please explain your answer.

29. Are there any potential barriers to developing and implementing an antibiogram in this facility?

30. Is there anything else you would like to say regarding this topic before we finish this interview?

Thank you very much for your time.
